# The effect of a continuing medical education program on Venous thromboembolism prophylaxis utilization and mortality in a tertiary-care hospital

**DOI:** 10.1186/1477-9560-12-9

**Published:** 2014-04-28

**Authors:** Fahad Al-Hameed, Hasan M Al-Dorzi, Essam Aboelnazer

**Affiliations:** 1 Department of Intensive Care, King Abdul-Aziz Medical City; College of Medicine, King Saud Bin Abdul-Aziz University for Health Sciences, National Guard Health Affairs, Jeddah, Saudi Arabia; 2 Saudi Association for Venous Thromboembolism (SAVTE), Jeddah, Saudi Arabia; 3 Department of Intensive Care, King Abdulaziz Medical City and King Saud Bin Abdulaziz University for Health Sciences, College of Medicine, Riyadh, Saudi Arabia; 4 Department of Surgery, Medical College, University of Um Al-Qura, Mekkah, Saudi Arabia

**Keywords:** Venous thromboembolism, Deep venous thrombosis, Pulmonary embolism, Thromboprophylaxis, Continuing medical education

## Abstract

**Background:**

Venous thromboembolism (VTE) prophylaxis is underutilized for hospitalized patients. The primary objective of this study was to assess the impact of a continuing medical education (CME) program on thromboprophylaxis and VTE-associated mortality in a tertiary-care hospital.

**Methods:**

This was a retrospective study of all patients admitted to a tertiary-care hospital from 01/07/2009 to 30/06/2010 (after a CME program that aimed at improving VTE prophylaxis) and had confirmed VTE during stay. VTE prophylaxis utilization and associated mortality were assessed in them and compared to those of a similar cohort of patients hospitalized in the previous 12 months.

**Results:**

There were 147 confirmed VTE cases in the study period (surgical: 26.5% and medical: 73.5%). Most (63.9%) VTE patients received prophylaxis after the CME program compared with 36.5% in the previous 12 months (relative risk 1.73; 95% confidence interval, 1.38-2.18; *P <* 0.001). More surgical (82.1%) than medical (57.4%) patients received prophylaxis (*P < 0.01*). VTE-associated mortality rate was 10.9% with a significant decrease after the CME program (relative risk, 0.52; 95% confidence interval, 0.30-0.90). This mortality was lower for those who received VTE prophylaxis compared to those who didn’t (4.3% and 22.6%, respectively; *P < 0.01*). Additionally, VTE-associated deaths represented 1.1% of total hospital mortality compared to 1.9% in the 12 months before CME program (relative risk, 0.58; 95% confidence interval, 0.32-1.04; *P* = 0.07).

**Conclusions:**

A CME educational program to improve VTE prophylaxis in a tertiary-care hospital was associated with improvement in VTE prophylaxis utilization and VTE-associated mortality. Such programs are highly recommended.

## Background

Venous thromboembolism (VTE) is a major cause of morbidity and mortality in hospitalized patients, accounting for 5-10% of in-hospital fatalities [1,2]. Although thromboprophylaxis has been proven to be effective [3,4], it continues to be underemployed. In the “Epidemiologic International Day for the Evaluation of Patients at Risk for Venous Thromboembolism in the Acute Hospital Care Setting” (ENDORSE) study, a multi-national and multi-center cross-sectional survey, only about half of hospitalized patients received thromboprophylaxis according to the 2004 American College of Chest Physicians (ACCP) guidelines [5].

The wide gap between VTE prophylaxis guidelines and practice represents a real challenge to the medical community. Many measures have been suggested to bridge this gap. These measures included periodic educational sessions to increase health care providers’ awareness of the necessity of thromboprophylaxis [6,7], incorporating thromboprophylaxis in medical admission order sets [8] and electronic alerts using computer-based clinical decision support systems [9]. However, the effectiveness of each of these measures is variable and may be questionable.

The primary objective of the current study was to assess the impact of a multifaceted continuing medical education (CME) program conducted in a tertiary-care hospital on the practices of VTE prophylaxis and on VTE-associated in-hospital mortality.

## Materials and methods

### Patients and setting

This was a retrospective observational study that was conducted at King Fahad General Hospital, a 900-bed tertiary-care university-affiliated hospital in Jeddah, Saudi Arabia. It was operated by the Saudi Ministry of Health and admitted medical and surgical patients. We had previously conducted a study in the same hospital to find out the rate of VTE prophylaxis in hospitalized patients according to the ACCP guidelines [10]. We found that for the 178 patients with confirmed VTEs that were diagnosed during hospitalization between July 1, 2008 and June 30, 2009, only 36.5% received VTE prophylaxis (44.1% for eligible surgical patients and 21.7% for medical patients) [10]. In this study and after a hospital-wide CME program, we evaluated all patients who were admitted to the hospital from July 1, 2009 to July 31, 2010 and had newly-diagnosed VTE that occurred during hospitalization. To identify these patients, we used the same methods of the previous study [10]. Hence, patients with ICD-9 codes related to VTE were identified from the hospital discharge data and reviewed. Only patients with confirmed VTE (deep vein thrombosis [DVT] or pulmonary embolism [PE] or both) were included. The clinical diagnosis of DVT was confirmed by extremity venous Doppler ultrasound and of PE by chest computed tomography or echocardiography in major PE and hemodynamic instability. During the study periods, there was no change in the hospital operation and patient mix. The Institutional Review Board of the hospital approved the current study and granted waiver of consent.

### The continuing medical education program

The CME program was intended to increase the hospital staff’s awareness of VTE burden and the value of VTE prophylaxis and was conducted in a large conference room on three consecutive days from June 22 to 24, 2009. Physicians of different specialties constituted the majority of attendees followed by registered nurses. The program consisted of didactic lectures, which were presented in English by physicians of different specialties and focused on VTE epidemiology, VTE burden in different medical and surgical specialties, VTE risk assessment, methods of VTE prophylaxis and the evidence-based VTE prophylaxis guidelines. During the program, a new paper-based VTE risk assessment tool [11] was introduced for physicians to voluntarily complete for newly-admitted patients. Moreover, pocket-size booklets summarizing VTE prophylaxis guidelines and handouts on how to administer low-molecular-weight heparin and provide mechanical prophylaxis were distributed to the program attendees. Additionally, reminder posters were thereafter continuously exhibited in the various hospital wards. An attending physician played the role of project champion advocating VTE prophylaxis throughout the study period. The CME program and VTE prophylaxis utilization were supported by the hospital leadership and administration.

### Data collection

We collected our data by reviewing the medical records and hospital computerized database in a similar way to our previous study [10]. The following information was noted for each patient with confirmed VTE: age, sex, admission category (medical versus surgical) and VTE risk factors (Table 1). VTE risk was categorized according to the Caprini Risk Assessment Model [11]. Additionally, we obtained statistics on all deaths that occurred in the hospital during the study period and on in-hospital deaths due to circulatory and respiratory collapse, which could be caused by PE. The primary outcomes of this study were VTE prophylaxis utilization (mechanical and/or pharmacological) as per the ACCP recommendations [12] before the diagnosis of VTE and VTE-associated mortality in the hospital.

**Table 1:** Baseline characteristics of patients with confirmed venous thromboembolism (July 1, 2009-June 30, 2010)

	**All patients**
	**N = 147**
Age* **(years), mean ± SD (range)**	56 ± 3.98 (52.9-67.5)
**Age classes, N (%)**	
< 40 years	1 (0.7)
40-60 years	112 (76.2)
> 60 years	31 (21.1)
**Male gender, N (%)**	84 (57.1)
**Risk factors, N (%)**	
Association with surgery	39 (26.5)
Immobilization	27 (18.4)
Smokers	37 (25.2)
Hypertension	107 (72.8)
Diabetes	92 (62.6)
Obesity	26 (17.7)
Indwelling venous device	58 (39.5)
History of infection	21 (14.3)
Inflammatory disease	77 (52.4)
Cancer	18 (12.4)
Chronic obstructive pulmonary disease	5 (3.4)
Respiratory failure	21 (14.3)
Varicose veins	16 (11.3)
History of stroke	45 (30.6)
History of heart failure	21 (14.3)
Myocardial infarction	65 (44.2)
Peripheral vascular disease	70 (47.6)
History of nephrotic syndrome	9 (6.1)

*Age was missing in 3 patients.

### Statistical analysis

Statistical analysis was performed using the Statistical Package for Social Sciences (SPSS for Windows, version 17). Descriptive statistics were reported as mean with standard deviation for continuous variables and as frequency with percentage for categorical variables. Differences between groups were tested using the Chi-square test. Whenever any of the expected values were less than 5, Fisher’s exact test was used instead. Comparison between previous study [10] results and current study was also performed. The risk of VTE prophylaxis and of VTE-associated in-hospital mortality after the CME program was compared to that before the program and was presented as relative risk with 95% confidence interval. All statistical tests used were two-tailed at a 5% level of significance.

## Results

Between July 1, 2009 and June 30, 2010, 147 cases were confirmed to have VTE during their hospitalization. Table 1 shows their baseline characteristics. Thirty nine (26.5%) VTE patients were surgical while 108 VTE events (73.5%) were associated with medical admissions. Most patients were > 40 years old and males (57.1%). DVT was diagnosed in 131 (89%) patients and 16 (11%) had DVT that progressed to PE.

### VTE risk categorization

Table 2 shows the VTE risk stratification of the cohort according to the 2008 ACCP guidelines [12]. Most (94.8%) surgical patients were classified as very high risk, 1 (2.6%) as high risk, and 1 (2.6%) as moderate risk. For medical patients, 65 (60.2%) patients were high risk and 43 (39.8%) were moderate risk.

**Table 2:** The relationship between venous thromboembolism prophylaxis and the mortality of medical and surgical patients

	**All patients**	**Medical patients**	**Surgical patients**	***P *** **value**
	**N = 147**	**N = 108**	**N = 39**	
VTE risk*, N (%)				
Very high risk	37 (25.2)	0 (0)	37 (95.9)	< 0.01
High risk	66 (44.9)	65 (60.2)	1 (2.6)	< 0.01
Moderate risk	44 (29.9)	43 (39.8)	1 (2.6)	< 0.01
**VTE prophylaxis provided, N (%)**	94 (63.9)	62 (57.4)	32 (82.1)	< 0.01
**Mortality, N (%)**	16 (10.9)	14 (13)	2 (5.1)	> 0.05
**Mortality in patients with VTE prophylaxis, N (%)**	4 (4.3)	3 (4.8)	1 (3.1)	> 0.05
**Mortality in patients without VTE prophylaxis, N (%)**	12 (22.6)	11 (23.9)	1 (14.3)	> 0.05

VTE, venous thromboembolism.

*VTE risk assessment performed according to Caprini Risk Assessment Model.

### Venous thromboembolism prophylaxis practices

**Figure 1:**
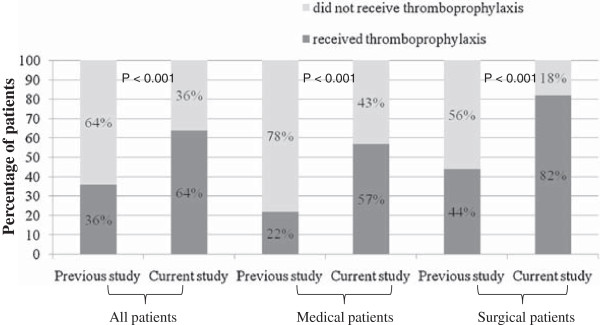
**Practice of venous thromboembolism prophylaxis before hospitalized patients developed deep vein thrombosis or pulmonary embolism.** This practice is described before (previous study, N = 168 patients) and after (current study, N = 147 patients) continuing medical education program.

Based upon the ACCP VTE prophylaxis guidelines, all of the 147 patients in the cohort were eligible to receive VTE prophylaxis. After the CME program, we found that 63.9% of the 147 patients who developed VTE during hospitalization received VTE prophylaxis. This represents a statistically significant improvement (relative risk 1.73; 95% confidence interval, 1.38-2.18; *P* < 0.0001) compared to the VTE prophylaxis rate (36.5%) in our previous study [10] (Figure 1).

The improvement in VTE prophylaxis after the CME program was observed in both surgical and medical patients (Figure 1). However, surgical patients continued to have higher VTE prophylaxis rates than medical patients (82.1% and 57.4%, respectively) (Table 2). VTE prophylaxis was most common in very high risk surgical patients (86.5%). Almost half (49.2%) of high risk medical patients and one third (32.6%) of moderate risk medical patients did not receive prophylaxis. We also found that more patients with DVT alone received VTE prophylaxis compared to those patients who had DVT progressing to PE (69.5% versus 26.3%; *P < 0.001*).

### Mortality

**Figure 2:**
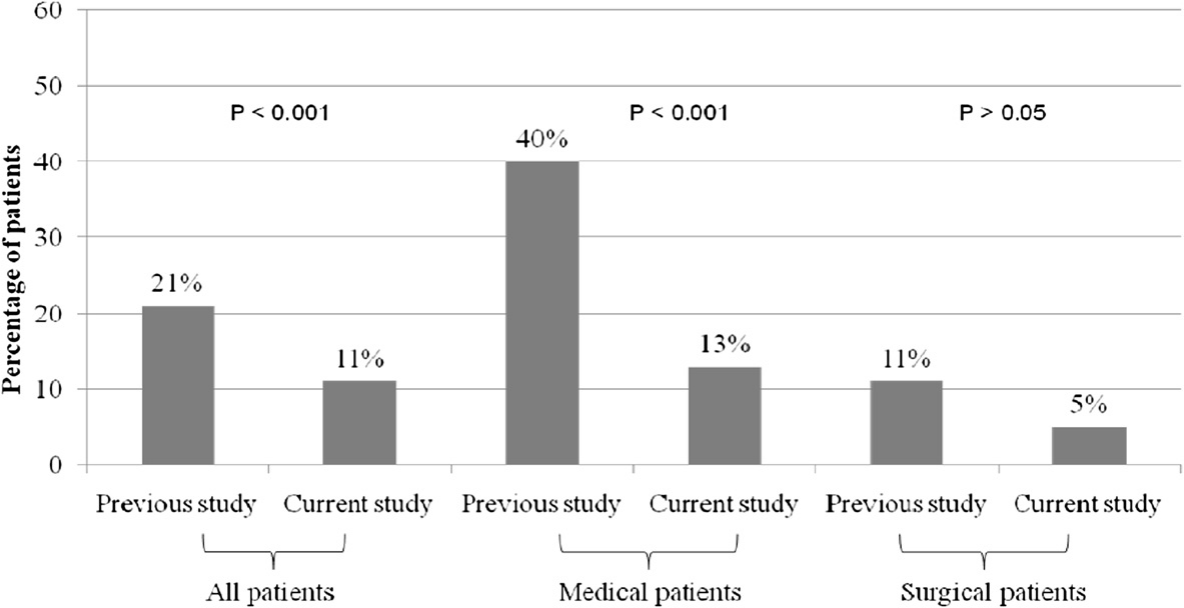
Venous thromboembolism-associated mortality before (previous study) and after (current study) a continuing medical education program.

In the current study, 16 (10.9%) patients who developed VTE during hospitalization died compared to 37 (20.8%) patients in the previous study (Figure 2). Hence, there was a significant decrease in VTE-associated mortality after the CME program (relative risk, 0.52; 95% confidence interval, 0.30-0.90; *P* = 0.02). The mortality rate in medical patients (13%) was not significantly different compared to that of surgical patients (5%) as shown in Table 2 (*P > 0.05)*. The mortality rate in medical patients was significantly lower in the current study compared to the previous study [10] (*P* < 0.001) (Figure 2). The decline in the mortality of surgical patients was not statistically significant (Figure 2).

In the current study, the mortality rate in the VTE patients who received prophylaxis was 4.3%, which is significantly lower than the mortality of those who did not receive it (22.6%; *P < 0.01)*. The mortality difference associated with VTE prophylaxis was significant in medical patients (*P* < 0.01) but not in surgical patients (P > 0.05). All deaths occurred in very high/ high VTE risk groups. Moreover, the mortality of patients with DVT progressing to PE (16%) patients was significantly higher than that of DVT patients (0%; *P < 0.001)*.

During the current study period, 1464 patients died in the hospital. The VTE-associated hospital mortality (N = 16) accounted for 1.1% of the total in hospital mortality. This is in contrast to 37 VTE-associated deaths (1.9% of 1968 hospital deaths) in the previous study before the CME program. Hence, the relative risk of VTE-associated death after the CME program was 0.58 (95% confidence interval, 0.32-1.04; *P* = 0.07). Nevertheless, there was no difference in the proportion of hospital deaths due to circulatory and respiratory collapse before (N = 500, 25.4%) and after (N = 389, 26.6%) the CME program (*P* = 0.46).

## Discussion

The main findings of this study were the following: 1- appropriate VTE prophylaxis rate for patients who developed VTE during hospitalization increased significantly from 36.5% to 63.9% after a hospital-wide CME program; 2- VTE prophylaxis utilization improved in both surgical and medical patients; 3- VTE-associated mortality was lower in patients receiving VTE prophylaxis and this mortality risk decreased after the CME program and 4- the proportion of hospital deaths associated with VTE tended to decrease after the CME program.

VTE frequently complicates acute illnesses that require hospitalization. However, VTE prophylaxis for hospitalized patients remains underutilized. This was observed in multiple studies [5,13-15]. In a prospective study of 5,451 consecutive patients with ultrasonographically confirmed DVT, only 42% of inpatients had received prophylaxis within 30 days before DVT diagnosis [15]. A cross-sectional survey that assessed the adherence to the 2004 ACCP VTE prophylaxis guidelines across 32 countries showed considerable variation among countries, with adherence to guidelines ranging from 0.2% to 92% (mean = 59%) for surgical patients and 3% to 70% (mean = 40%) for medical patients [5]. In our previous study in the same hospital, we found that VTE prophylaxis was frequently missed in patients who eventually developed VTE [10]. With the global recognition of the gap between VTE prophylaxis guidelines and real practice, different methods were suggested to improve VTE prophylaxis utilization with variable effectiveness. In a before-after study in a community hospital, the administration of order sets that were used voluntarily by internists was associated with an increase in the percentage of patients who were ordered prophylaxis (44.0% versus 20.6% for patients admitted with free text orders; *P* < 0.0001) [8]. Additionally, the hospital-wide DVT prophylaxis for medical inpatients increased from 12.8% to 25.8% of patient-days (*P* < 0.0001) [8]. Kucher et al. randomized 1,255 patients to an intervention group, in which the responsible physician received a computer alert of the patient’s VTE risk, and 1,251 patients to a control group, in which no alert was issued, and found that more patients in the intervention group received mechanical prophylaxis (10.0% versus 1.5%; *P* < 0.001) and pharmacologic prophylaxis (23.6% versus 13.0%; *P* < 0.001) [9]. The computer alert reduced VTE risk at 90 days by 41% (hazard ratio, 0.59; 95% confidence interval, 0.43-0.81; *P* = 0.001) [9]. The effect of CME programs on VTE prophylaxis has also been evaluated. Anderson et al. conducted a cluster randomized trial that evaluated the effects of CME with or without quality assurance on VTE prophylaxis in 3,158 high-risk medical patients and found significant increases in the proportion of patients receiving prophylaxis in all hospitals, including the control hospitals, over the study period (from 29% in 1986 to 52% in 1989; *P <* 0.001) [6]. The increase was greater in hospitals that had CME with or without quality assurance than the control hospitals (+28% versus +11%; *P <* 0.001) [6]. A systematic review of randomized controlled trials found that continuing education meetings with or without other interventions had only small effect on the clinical practice of healthcare professionals or healthcare outcomes [16]. Additionally, the effect is probably temporary. In our study, we have found that VTE prophylaxis in patients who developed VTE during hospitalization was significantly higher in the one year after a hospital-wide CME program with the increase observed in both surgical (from 44.1% to 82.1%) and medical (from 21.7% to 57.4%) patients.

Multifaceted strategies have been shown to be more effective than any single approach in improving VTE prophylaxis in hospitals. Scaglione et al. found that the implementation of a multi-strategy approach made up of educational presentations, pocket guidelines, the implementation of a working group to identify barriers to change and the introduction of risk-reminder cards in an Italian teaching hospital increased the appropriate use of VTE prophylaxis in surgical patients from 64% to 97% [17]. Cohn et al. demonstrated that the implementation of a multifaceted VTE prophylaxis quality improvement program that combined regular education, dissemination of a decision support tool and regular audit-and-feedback to resident physicians in a US hospital resulted in an increase in appropriate VTE prophylaxis from 43% to 68% after 12 months and to 85% after 18 months [18]. At an Australian hospital, Gallagher et al. evaluated a multifaceted approach and observed an increase in the VTE risk assessment in the ward setting (7.7% to 100%; *P* < 0.001), an increase in the proportion of patients receiving anticoagulant prophylaxis (48% to 74%; *P* = 0.01) and a reduction in the annual VTE rate (relative risk, 0.68; 95% confidence interval, 0.47-0.99; *P* = 0.04) [19]. In a study conducted in Saudi Arabia, a quality improvement project over 14 months consisting of education of physicians, development of a VTE prophylaxis protocol, weekly monitoring of compliance, recommending VTE prophylaxis during the multidisciplinary rounds and feedback whenever a deviation from the protocol occurred, was associated with improvement in VTE prophylaxis utilization from 63% in the initial project stage to 100% at the final stage (overall rate = 91%) [20]. A systematic review of studies performed between 1996 and 2003 that focused on strategies to improve VTE prophylaxis practices observed that a passive dissemination of guidelines was associated with poor adherence to both the guidelines and the provision of adequate prophylaxis, and multiple strategies were more effective than any single strategy [7]. Our CME program included didactic lectures, distribution of VTE prophylaxis guidelines, handouts on how to use low-molecular-weight heparin and to provide mechanical prophylaxis and reminder posters. It was also associated with hospital leadership support, voluntary VTE risk assessment and the presence of a champion that advocated VTE prophylaxis. This multifaceted approach was probably behind the program success.

VTE prophylaxis has been shown in multiple studies to reduce DVT and PE [3,4,21]. However, studies on the relationship between VTE prophylaxis and mortality have shown mixed results. A metaanalysis based on old studies in general surgery patients found that low-dose unfractionated heparin compared with no thromboprophylaxis or placebo was associated with decrease in all-cause mortality from 4.2 to 3.2% [22]. Missemiti et al. found that low-molecular-weight heparin versus placebo or no treatment was associated with a trend toward lower mortality (relative risk, 0.54; 95% confidence interval, 0.27-1.10) [4]. A recent metaanalysis of studies in nonsurgical patients found that VTE prophylaxis did not reduce mortality (odds ratio, 0.94; 95% confidence interval, 0.84-1.04) [21]. In the current study, patients who received prophylaxis had lower VTE-associated death than those who did not receive prophylaxis (4.3% and 22.6%, respectively). This observation is interesting and suggests that VTE prophylaxis may have led to lower mortality in patients who eventually developed VTE. However, VTE prophylaxis may reflect better care in general with it being only one component among other interventions. Additionally, VTE-associated mortality accounted for 1.1% of the total hospital mortality (June 2009-July 2010) compared to 1.9% in the previous 12 months [10]. This decrease approached statistical significance (*P* = 0.07) and might be related to the CME program.

Our study has some limitations. It is a retrospective study performed at a single center. We evaluated VTE prophylaxis utilization only in patients who developed VTE. However, we believe that the observed improvement in VTE prophylaxis in these patients probably reflects a practice improvement in the other patients after the CME program. The unavailability of the total number of patients admitted to the hospital resulted in the inability to provide incidence statistics. We assumed that at least a proportion of the deaths after sudden circulatory and respiratory collapse were due to undiagnosed fatal PE. This is because post-mortem autopsy is very seldom performed in Saudi Arabia for cultural reasons. Additionally, we evaluated the effect of the educational program in one-year period. There may have been variation in VTE prophylaxis utilization during that year and we do not know if the improvement was sustained in the subsequent years. We acknowledge that the sustainability of any quality improvement project should be considered at the initial design process [23], which requires leadership engagement, staff involvement and continuing training to sustain the change processes. Moreover, we did not assess bleeding events after hospitalization, which could have increased after VTE prophylaxis.

## Conclusions

In conclusion, a hospital-wide multifaceted CME program that aimed at improving healthcare providers’ VTE awareness and prophylaxis practices was associated with significant improvement in VTE prophylaxis practices, reflected by increased VTE prophylaxis utilization in patients who developed VTE during hospitalization. This was also associated with decrease in the mortality of these patients. Such a multifaceted program is recommended for healthcare professionals working in acute care hospitals.

## Abbreviations

ACCP: American College of Chest Physicians; CME: Continuing medical education; DVT: Deep vein thrombosis; PE: Pulmonary embolism; VTE: Venous thromboembolism.

## Competing interests

The authors received honoraria from Sanofi-Aventis, KSA for presenting in continuing medical education programs.

## Authors’ contributions

FH participated in conception and design, participated in interpretation of data, critically revised the manuscript for important intellectual content and approved the final version to be published. HD participated in analysis and interpretation of data, helped to draft the manuscript, critically revised the manuscript for important intellectual content and approved the final version to be published. EA participated in conception and design, helped in interpretation of data, critically revised the manuscript for important intellectual content and approved the final version to be published. All authors read and approved the final manuscript.
